# Changes needed in health information systems to support an ageing population

**DOI:** 10.2471/BLT.25.294184

**Published:** 2025-12-02

**Authors:** Ana Mendez-Lopez, Kidong Park, Roland D Hensman

**Affiliations:** a World Health Organization Regional Office for the Western Pacific, Division of Data, Strategy and Innovation, United Nations Avenue, 1000 Metro Manila, Manila, Philippines.

## Abstract

Health information systems are essential for evidence-based decision-making and must transform to support healthy ageing in rapidly ageing societies. This change requires strengthening of the structures and processes that enable the collection, integration, analysis and use of comprehensive, integrated and person-centred information in health, social and long-term care sectors. Interoperability and governance mechanisms are important to link data and services and ensure coordinated and continuous care for older adults. Health information systems should incorporate ageing-relevant areas such as functional ability, well-being, end-of-life preferences, living environments, informal and formal caregiving, and social determinants of health. Advanced analytics and tools to support decision-making can provide actionable insights to improve the quality of care, evaluate progress and inform the design of age-friendly policies and services. Equally, systems must be user-centred and accessible, adapted to varying levels of digital literacy, and co-designed with older adults and communities. Engaging older adults and communities in the design of health information systems can enhance relevance, equity, trust and uptake. Such systems can support older adults to actively manage their health. Finally, robust ethical and legal frameworks are essential to guide the responsible design, implementation and use of health information systems. These frameworks should safeguard privacy and uphold dignity, particularly concerning sensitive data, informed consent and decision-making capacity in later life.

## Résumé

Les systèmes d’information sur la santé sont essentiels à la prise de décisions fondées sur des données scientifiques. Ils doivent évoluer pour soutenir le vieillissement en bonne santé dans des sociétés qui connaissent une augmentation rapide de l’âge moyen des habitants. Ce changement nécessite de renforcer les structures et processus qui permettent la collecte, l’intégration, l’analyse et l’utilisation d’informations complètes, intégrées et centrées sur les personnes dans les secteurs de la santé, des services sociaux et des soins de longue durée. L’interopérabilité et les mécanismes de gouvernance sont essentiels pour relier les données et les services et garantir des soins coordonnés et continus aux personnes âgées. Les systèmes d’information sur la santé doivent intégrer des domaines liés au vieillissement tels que les capacités fonctionnelles, le bien-être, les préférences de fin de vie, les cadres de vie, les soins informels et formels, ainsi que les déterminants sociaux de la santé. Des analyses et des outils avancés destinés à soutenir la prise de décisions peuvent fournir des renseignements exploitables pour améliorer la qualité des soins, évaluer les progrès et éclairer la conception de politiques et de services adaptés aux personnes âgées. De même, les systèmes doivent être centrés sur l’utilisateur et accessibles, adaptés aux différents niveaux de maîtrise des outils numériques et conçus en collaboration avec les personnes âgées et les groupes de populations associés. La participation de personnes âgées et de groupes de populations dans la conception des systèmes d’information sur la santé permet d’améliorer leur pertinence, leur équité, la confiance qu’ils inspirent et leur adoption. De tels systèmes sont de nature à aider les personnes âgées à gérer leur santé de manière active. Enfin, des cadres éthiques et juridiques solides sont essentiels pour guider la conception, la mise en œuvre et l’utilisation responsables des systèmes d’information sur la santé. Ces cadres doivent protéger la confidentialité et préserver la dignité, en particulier en ce qui concerne les données sensibles, le consentement éclairé et la capacité des personnes âgées à prendre des décisions.

## Resumen

Los sistemas de información sanitaria son esenciales para la toma de decisiones basada en la evidencia y deben transformarse para apoyar el envejecimiento saludable en sociedades que envejecen rápidamente. Este cambio requiere fortalecer las estructuras y los procesos que permiten la recopilación, integración, análisis y uso de información completa, integrada y centrada en la persona en los sectores de atención sanitaria, social y de cuidados a largo plazo. La interoperabilidad y los mecanismos de gobernanza son fundamentales para vincular datos y servicios y garantizar una atención coordinada y continua para las personas mayores. Los sistemas de información sanitaria deberían incorporar áreas relevantes para el envejecimiento, como la capacidad funcional, el bienestar, las preferencias al final de la vida, los entornos habitacionales, los cuidados informales y formales, y los determinantes sociales de la salud. Los análisis avanzados y las herramientas de apoyo a la toma de decisiones pueden ofrecer información útil para mejorar la calidad de la atención, evaluar los progresos e informar el diseño de políticas y servicios adaptados a las personas mayores. Asimismo, los sistemas deben ser accesibles y centrados en el usuario, adaptados a los distintos niveles de alfabetización digital y codiseñados con las personas mayores y las comunidades. Involucrar a las personas mayores y a las comunidades en el diseño de los sistemas de información sanitaria puede mejorar la pertinencia, la equidad, la confianza y la adopción. Dichos sistemas pueden ayudar a las personas mayores a gestionar activamente su salud. Por último, los marcos éticos y jurídicos sólidos son esenciales para orientar el diseño, la implementación y el uso responsables de los sistemas de información sanitaria. Estos marcos deben salvaguardar la privacidad y preservar la dignidad, especialmente en lo relativo a los datos sensibles, el consentimiento informado y la capacidad para tomar decisiones en etapas avanzadas de la vida.

## ملخص

تعد نظم المعلومات الصحية أمرًا حيويًا لاتخاذ القرارات القائمة على الأدلة، ويجب تطويرها لدعم حدوث الشيخوخة بشكل صحي في المجتمعات التي تشهد تسارعًا في الشيخوخة. يتطلب هذا التغيير تعزيز الهياكل والعمليات التي تتيح جمع ودمج وتحليل واستخدام معلومات شاملة ومتكاملة ومرتكزة على الفرد، في قطاعات الصحة والرعاية الاجتماعية والرعاية طويلة الأجل. وتُعدّ آليات التشغيل البيني والحوكمة مهمةً لربط البيانات والخدمات، وضمان رعاية منسقة ومتواصلة لكبار السن. وينبغي أن تُدمج نظم المعلومات الصحية الجوانب ذات الصلة بالشيخوخة، مثل القدرة الوظيفية، والرفاهية، وتفضيلات نهاية العمر، والبيئات المعيشية، والرعاية الرسمية وغير الرسمية، والمحددات الاجتماعية للصحة. ويمكن للتحليلات والأدوات المتقدمة لدعم اتخاذ القرارات أن تُقدّم رؤى عملية لتحسين جودة الرعاية، وتقييم التقدم المُحرز، وتوجيه تصميم السياسات والخدمات التي تضع كبار السن في الاعتبار. وبالمثل، يجب أن تكون الأنظمة مرتكزة على المستخدم، ويسهل الحصول عليها، ومتوافقة مع مستويات متباينة من المعرفة الرقمية، ومُصمّمة بالتعاون مع كبار السن والمجتمعات المحلية. إن إشراك كبار السن والمجتمعات المحلية في تصميم أنظمة المعلومات الصحية يمكنه تعزيز مدى الملاءمة، وتحقيق العدالة، والثقة، وزيادة الإقبال عليها. ويمكن لهذه الأنظمة أن تدعم كبار السن في رعاية صحتهم بفعالية. وأخيرًا، تُعد أطر العمل الأخلاقية والقانونية الفعالة، أمرًا حيويًا لإرشاد التصميم والتنفيذ والاستخدام المسؤول لأنظمة المعلومات الصحية. وينبغي أن تحمي هذه أطر العمل هذه الخصوصية وتحفظ الكرامة، وخاصة فيما يتعلق بالبيانات الحساسة، والموافقة المستنيرة، والقدرة على اتخاذ القرارات في مراحل متقدمة من العمر.

## 摘要

卫生信息系统是循证决策制定的关键所在，且该系统必须进行相应变革方能支持快速老龄化社会实现健康老龄化。如要推动卫生信息系统变革，必须先强化能促进健康、社会和长期护理行业内全面、整合和以人为本信息的收集、整合、分析和使用的相关结构和流程。互操作性和治理机制对链接数据和服务以及确保为老年人持续提供协调护理服务而言非常重要。卫生信息系统应涵盖老龄化相关领域，例如功能能力、福祉、临终偏好、生活环境、非正规和正规照护以及健康问题社会决定因素。运用高级分析法和工具来支持决策制定，以提供可采取行动的深刻见解，从而改善护理质量、评估进展并了解如何设计老年友好型政策和服务。同样重要的一点是，此类系统必须以用户为中心且易于使用，适用于拥有不同数字素养水平的用户，并邀请了老年人和社区进行共同设计。老年人和社区参与卫生信息系统的设计，有助于提升相关性、公平性、信任度和采纳率。此类系统可支持老年人积极管理自身健康。最后，稳健的伦理和法律框架对于指导以负责任的方式设计、实施和使用卫生信息系统而言至关重要。这些框架应可保护隐私和维护尊严，特别是在与敏感数据、知情同意和晚年决策能力相关的方面。

## Резюме

В контексте стремительного старения населения реформирование информационных систем здравоохранения является крайне важным условием для принятия решений, основанных на доказательной базе, а также эффективной поддержки здорового старения. Такое изменение требует укрепления структур и процессов, которые делают возможным сбор, интеграцию, анализ и использование всеобъемлющей, объединенной и человекоориентированной информации в секторах здравоохранения, социального обслуживания и долгосрочного ухода. Механизмы взаимодействия и управления важны для объединения данных и услуг, а также для обеспечения скоординированной и непрерывной помощи пожилым людям. Информационные системы здравоохранения должны включать данные из областей, связанных со старением, такие как сведения о функциональных способностях, общем благополучии, предпочтениях в отношении ухода в конце жизни, условиях проживания, а также о неофициальном и официальном уходе и социальных детерминантах здоровья. Развитые аналитические методы и средства поддержки принятия решений могут предоставить полезную информацию, которая позволит улучшить качество медицинского обслуживания, оценивать прогресс и обеспечивать информационное наполнение при разработке политик и услуг, благоприятствующих пожилым людям. Равным образом эти системы должны быть ориентированы на пользователя и доступны, адаптированы к различным уровням цифровой грамотности, и к их разработке следует привлекать пожилых людей и общественность. Привлечение пожилых людей и общественности к разработке информационных систем здравоохранения может повысить релевантность, равенство возможностей, доверие и охват услугами. Такие системы могут содействовать пожилым людям в активной заботе о своем здоровье. Наконец, надежные этические и юридические рамки крайне необходимы для того, чтобы координировать ответственную разработку, осуществление и использование информационных систем здравоохранения. Эти нормы должны защищать конфиденциальность и соблюдать достоинство пользователей, в частности относительно конфиденциальных данных, осознанного согласия и способности к принятию решений на склоне лет.

## Introduction

Population ageing is one of the most defining sociodemographic trends of the 21st century. Many countries are experiencing or approaching a demographic transition characterized by declining fertility and reductions in premature mortality, resulting in older population structures. These shifts bring considerable social, economic and health-system implications. Ageing societies face changing epidemiological profiles, including a higher burden of noncommunicable diseases, injuries and multimorbidity, alongside increased demand for long-term care, integrated services and social protection.^1^^,^^2^

As countries adapt to these demographic changes, strengthening and transforming health information systems becomes increasingly important. Health information systems are a cornerstone of evidence-based policy-making in health and are defined by the World Health Organization (WHO) as, “systems that collect data from health and other relevant sectors, analyse the data and ensure their quality, relevance, and timeliness, and convert them into information for health-related decision-making.”^3^ Health information systems integrate four key functions: data generation, compilation, analysis and synthesis, and communication and use.^3^ These functions have multiple components, including data sources, infrastructure, standards and interoperability frameworks, digital platforms, governance mechanisms, stakeholders, and processes for data use.^4^^,^^5^ Within this broad framework, health management information systems, such as facility-based and routine systems, which are designed to support planning, management and decision-making in health facilities and organizations, are one component of the overall structure of health information systems.^3^ Globally, health information systems are evolving to incorporate new technologies, improve data quality, timeliness, analytics and visualization, and expand coverage to traditionally underserved populations and settings. Increasingly, health information systems also need to adapt to complex social realities that intersect with health, such as climate change and increased migration and displacement.^6^^,^^7^

Healthy ageing as defined by WHO is, “the process of developing and maintaining the functional ability that enables well-being in older age.”^2^ In this context, health information systems must move beyond a narrow focus on disease management to support policies and systems that promote well-being in later life, preserve functional ability and ensure equitable access to care and social support for older adults, thereby accelerating action towards the implementation of the United Nations Decade of Healthy Ageing.^8^ This expansion requires adapting health information systems to systematically capture, analyse and use health and related data on older populations. These data are not only useful for policy-makers and service providers, but are also meaningful to older adults, their families and their communities. Health information systems adapted to the goals of healthy ageing should help generate evidence for policy-making, improve the quality and responsiveness of health and social services, and empower older adults to actively manage their health.

To achieve these goals, an ageing-relevant approach must be used in adapting health information systems in several key functional areas, such as inclusive data collection, user-centred design, system integration, decision support, community engagement, privacy protection and evaluation frameworks (Box 1). While these areas align with international standards for health information systems, tailoring them to the specific needs of ageing populations is essential to fully support healthy ageing.

Box 1 **Key components of the health information system adapted to support healthy ageing**

**Comprehensive data beyond the traditional health sector**

- Includes data from health, social and long-term care sectors
- Registers care facilities and professionals, including designated informal caregivers
- Captures functional ability, well-being, social determinants and end-of-life preferences
- Incorporates data from digital tools (e.g. wearable devices) and geriatric assessments

**Accessibility and user-centred design**

- Provides multiuser access tailored to different roles and responsibilities
- Designs user interfaces adapted to the digital literacy levels of providers, caregivers and older adults
- Ensures usability and inclusiveness through active co-design with older adults and communities
- Includes low-tech and offline options to ensure access regardless of digital capacity

**Interoperability and integration**

- Links data across health, social and long-term care systems
- Ensures interoperability of electronic records across settings through shared identifiers and standards
- Enables real-time data exchange (for example, wearable devices and environmental data) to trigger alerts and preventive actions during events affecting older adults

**Advanced analytics and decision support**

- Develops ageing-relevant indicators and predictive analytic methods
- Combines longitudinal, routine and wearable-device data for risk prediction and personalized care planning
- Evaluates age-friendly policies, services and environments
- Builds workforce capacity in ageing-related information analysis and use

**Community and social engagement**

- Engages older adults, caregivers and communities in co-design and evaluation of health information systems
- Promotes digital literacy through training on use of digital tools and information
- Implements feedback mechanisms to ensure information is accessible, relevant and actionable for older adults and caregivers
- Strengthens accountability and trust through participatory governance and transparent information sharing

**Ethical, legal and privacy considerations**

- Embeds ethical and legal governance across systems to uphold dignity, autonomy and the rights of all older adults
- Documents end-of-life preferences and legal representatives within care systems
- Defines and enforces access permissions to balance data sharing, privacy and consent for users and caregivers
- Includes safeguards to prevent bias and misuse in digital and artificial intelligence systems

In this paper, we present a global perspective on transforming health information systems to support healthy ageing. We generate insights from technical products, reports and peer-reviewed literature identified through a purposive review to outline general principles applicable to different health systems. While these principles are broadly relevant, their implementation will depend on country context, institutional capacity and available resources.

## Comprehensive data

Comprehensive data are the backbone of health information systems that support healthy ageing. In aged and ageing societies, collecting such data requires expanding traditional health information to incorporate information from across the continuum of care, including health, social and care services. Data collection processes should consider that some older people may not access health care in a traditional way, as they live in long-term care facilities where data are not captured as part of the health information system. Health and well-being data from different care settings, such as home care or nursing homes, and social services must be systematically collected to reflect the different care environments in which older adults live.

### Registers and health records

Health information systems for the health workforce should be strengthened to include health and social caregivers and other allied health professionals relevant to ageing societies in health workforce registries. These changes should be supported by harmonized definitions and standards.^9^ Data on informal caregivers should also be captured and, along with data of other groups of health workers, be linked to patient records to improve continuity and coordination of care.^10^ Patient records should include information on family structures and designated caregivers, which helps support more holistic and person-centred care planning. Enhanced health information systems on human resources for health can help assess the densities and shortages of the health workforce so as to meet the unique needs of older adults in the context of increased demand for specialized geriatric care.

Facility registers should also be broadened to include public and private long-term care facilities, as well as social services. This information is especially important in countries with deregulated markets and a public–private mix in health services and social care for older people, where it can be difficult to register and monitor all facilities effectively.^11^ Furthermore, health facility catchment mapping should also include specialized institutions for care of older people, in the same way catchments are defined for schools, to ensure population needs are fully captured and services are adequately planned, coordinated and integrated.

Patient records and registers should be adapted to record end-of-life preferences, such as the desired place of care and death, preferred medical interventions (for example, resuscitation and intensive care), designated decision-makers and relevant cultural or spiritual practices. In countries where legally permitted, this information may also include preferences on medical assistance in dying, in accordance with national laws and ethical guidelines. These elements could be recorded in individual medical and patient records, or specialized palliative or long-term care registries, to ensure they are accessible to care providers.

In parallel, mortality data should be strengthened by systematically including deaths that occur in long-term care and care facilities for older people. This information will provide a fuller picture of mortality patterns in ageing populations and improve the completeness of vital statistics. Verbal autopsy processes should include immediate caregivers in long-term and care facilities for older people, as well as informal caregivers, as key informants. Their inclusion could help more accurately capture deaths in older people that occur outside of health facilities, particularly where medical certification of cause of death is incomplete or absent. Such information will strengthen the overall quality and completeness of mortality data within health information systems to better inform policies for healthy ageing.

### Data and indicators

Health information systems must also support the routine collection and monitoring of indicators relevant to healthy ageing. Functional ability, intrinsic capacity and well-being indicators should be regularly monitored using standardized definitions and routine geriatric health assessments, including those indicators recorded by digital tools.^12^^–^^15^ Data from wearable technology and remote monitoring systems, particularly devices that can detect accidents or emergencies, offer additional opportunities to monitor health status and trigger timely responses, especially in residential care settings and for older adults living alone.

Health information systems for healthy ageing must reflect the broader social realities that shape the lives of older adults. Thus, data on the social determinants of health and the ability to meet basic needs in older people should be systematically collected. This information includes socioeconomic status, housing, access to community resources and the built environment. These factors play an important role in shaping health trajectories in later life and can guide the development of programmes for age-friendly cities and communities.^12^^,^^16^^,^^17^ Loneliness and social isolation are important components of the well-being of older adults and are directly linked to mental and physical health outcomes, highlighting the need for health information systems to capture indicators on social connectedness, mental health and community participation.^12^^,^^18^^,^^19^

Measurement of ageism should also be incorporated into health information systems to ensure the well-being of older adults, as ageist attitudes and experiences negatively affect physical and mental health.^12^^,^^20^ Tools such as the WHO ageism scale provide a standardized approach to capturing these data to combat ageism and monitor progress towards more inclusive, age-friendly societies.^21^

To build a comprehensive evidence base for healthy ageing, these indicators must be complemented by longitudinal data that capture individual experiences and needs over time. Health information systems should therefore continue to promote the integration of life-course data that assess trajectories of healthy ageing, including longitudinal surveys focused on older populations. Cohorts based on administrative and routinely collected data can also offer a viable and sustainable alternative in resource-constrained contexts where traditional longitudinal studies or technologies are costly or difficult to maintain. Incorporating these data sources could enable the design of more targeted and equitable interventions to ensure healthy ageing for all.

### Interoperability and integration

Health information systems should be designed to enable seamless data flow across the full range of care settings and social services relevant to older adults. Such designs should integrate information from health facilities, long-term care providers and social services to ensure coordinated, integrated and person-centred care. WHO defines interoperability as, “the ability of different applications to access, exchange, integrate and use data in a coordinated manner through the use of shared application interfaces and standards, within and across organizational, regional and national boundaries, to provide timely and seamless portability of information and optimize health outcomes.”^3^ Interoperability standards should facilitate data exchange not only within the health sector but also between health and social care systems, enabling caregivers and service providers to work from a shared understanding of a person’s health and support needs. Integration of data systems across sectors could be centralized in electronic patient records that include linkage of records beyond health facilities and are operable in different settings through shared unique individual identifiers.

In addition to this structural integration, health information systems should also enable real-time data exchange across different sources as part of an integrated public health intelligence system, to enhance timely and coordinated action that supports healthy ageing through comprehensive and person-centred care. For example, live integration of data from wearable devices that detect accidents and sudden changes in vital signs can automatically feed into emergency response dashboards and other platforms used by community care services. This action would trigger alerts and enable timely responses to dispatch services or notify caregivers. Integration with digital home monitoring devices, electronic medication dispensers or telehealth systems could further support early identification of health deterioration or missed treatments among older adults.^22^ Such functions would be especially valuable in residential care settings and for older adults living alone. Similarly, linking health records with weather and meteorological information systems, such as environmental monitoring platforms, could prompt health and social care providers to activate preventive actions and responses during extreme weather events, such as heat waves and poor air quality. These events tend to disproportionately affect older adults, particularly frail older people.^23^^–^^25^

### Analytics and decision support

To meet the complex needs of ageing populations, health information systems must go beyond data collection to enable actionable insights that incorporate a healthy ageing approach. Advanced analytics encompass methods such as predictive modelling, data mining, machine learning, natural language processing and other computational techniques. These methods can generate actionable insights for healthy ageing. For instance, they can enhance tools such as geriatric assessment scores by integrating different data sources and identifying patterns that may not be evident through traditional analyses. In doing so, they provide a more comprehensive view of the health and well-being of older adults, and can help clinicians and care providers monitor changes over time and tailor interventions accordingly.

Building on these methods, different data sources, such as longitudinal surveys, wearable devices, remote monitoring systems and administrative cohorts based on routinely collected data, could further enhance awareness and risk prediction, especially when combined with advanced analytic models. Artificial intelligence (AI), which uses algorithms or systems that can learn from data and improve their performance over time, is a particularly powerful tool that can be used to identify early signs of functional decline, predict hospitalization risks and support personalized care planning through the analysis of longitudinal and real-time data.^26^

Additionally, population-level health analysis should systematically include standardized age-disaggregated data in relevant and sufficiently detailed groupings (for example, 5-year age brackets). The groupings should reflect biological, developmental and common societal factors, as well as age-specific considerations for disease prevention such as immunization schedules, screening recommendations and age-related risk profiles.^27^ Additional disaggregation by sex and other characteristics of older adults can support an intersectional analytical approach that recognizes the complex and multifaceted realities of older adults. Analytic approaches that combine population-based and health-facility data to estimate, for example, effective coverage, can further enrich such analyses by linking information on service provision with health outcomes of the population.^28^ Methods for linking household and provider data, for instance, have demonstrated how combining these sources improves understanding of whether care needs are effectively met across population groups.^28^ These finer insights into population health could, in turn, better inform age-specific policies and actions for health and social care.

Moreover, key analytic areas should include evaluating policies and interventions aimed at creating environments that enable healthy ageing.^29^^–^^31^ Such analyses assess how health, social and environmental policies affect functional ability, well-being and equity in later life. Analytical approaches could, for example, examine the impact of housing, transport, social protection and community engagement programmes on the health trajectories of older adults. These evaluations could generate important evidence to guide integrated strategies that address the physical, social and environmental determinants of health in later life, particularly for populations experiencing disadvantage.

Efforts to develop analytics that support healthy ageing should be accompanied by initiatives to build national capacity in this area. Building capacity requires training the health workforce, such as health workers, community health workers, long-term care staff, epidemiologists and data analysts, in collecting, analysing, using and communicating ageing-related information across different levels of care and decision-making. Such training could promote a shared understanding of how ageing-related information can inform practice and policy.

### Accessibility

As health information systems become more digitally advanced, accessibility and user-centred design are important to ensure that systems are usable and practical for all stakeholders, including older adults and their families, health workers, formal and informal care providers and social services personnel. Systems should be designed with different users in mind and allow multiple levels of access depending on roles and responsibilities.

Health and social care workers must have secure access to patient records, with functions adapted to their scope of work. These adapted functions include both limited and advanced interfaces that balance usability with safeguards for privacy and confidentiality. Features that support caregivers, such as alerts, shared care plans or simplified data entry tools, could enhance their ability to deliver timely and appropriate care.

Simplified interfaces should be designed with input from older adults to accommodate varying levels of digital literacy, so that digital transformation of health systems does not leave older adults or their caregivers behind. As older adults may have less digital skill, input based on their user experience could contribute to the design of simplified interfaces and intuitive navigation which allow older adults and their caregivers to access and benefit from their health data.^32^

While older adults may not directly input administrative or clinical data into health information systems, their ability to access and understand personal health information, such as test results, appointments or treatment summaries, could encourage their engagement in their care and shared decision-making.^32^^,^^33^ Importantly, they can also contribute to co-designing and testing the user experience of digital tools and reporting interfaces, which will help ensure that systems are intuitive, inclusive, and aligned with their needs and capacities. Additionally, automated data-sharing methods such as digital wallets could further support accessibility by simplifying how personal health information is stored and shared across providers, and catering to varying individual digital capabilities.

### Community and social engagement

Designing health information systems that support healthy ageing requires collaboration with older adults. When older people, health workers, social caregivers and community organizations are actively engaged in the co-design and evaluation of health information systems, these systems are more likely to be inclusive, relevant and grounded in real-life experiences.^32^ Such participatory processes can also help shape how information is presented and accessed, ensuring that digital tools and reporting systems are understandable and usable by people with different levels of digital literacy. In ageing societies, participatory approaches could help ensure that systems reflect the needs and priorities of the population, facilitate the uptake and quality of care, including through digital resources, and improve health outcomes.^32^^,^^33^

Health information systems should promote digital literacy and build capacity in older adults and caregivers. Such capacity-building could be supported through resources and training on using digital tools, accessing their health information and understanding how their data contribute to broader care and health outcomes.^34^^–^^36^ These systems should not only collect information about communities, but also serve as platforms for engaging with them. This engagement will reinforce social connectedness and enable older adults to participate actively in decisions about their care and the systems that support them.

Community engagement could also strengthen accountability and help build trust in digital health systems. Feedback mechanisms should be in place to ensure that the data collected through health information systems is returned to communities in formats that are useful, accessible and easy to understand. In turn, this feedback could empower individuals to actively manage their health and advocate for improved services.^34^ Moreover, involving different groups of older adults in system design could help ensure that health information systems are accessible and responsive to their educational, socioeconomic and cultural contexts, thereby advancing health equity and inclusion.

### Ethical considerations

Population ageing also requires health information systems to include ethical, legal and privacy considerations in various ways. First, as health information systems expand to cover increasingly sensitive aspects of people’s lives, including functional status and end-of-life preferences (as defined earlier), ethical and privacy safeguards become a priority. Second, given varying levels of digital literacy and cognitive ability, increased digitalization of health information systems poses ethical challenges for individual informed consent. Third, data access by an increased number of parties, from social services to informal caregivers, requires greater legal and privacy considerations. Therefore, it is imperative that ethical governance frameworks guide the design, implementation and use of these systems to uphold the rights and dignity of all older adults.

As such, health information systems must respect individual choices, especially for older people with significant loss of capacity who may rely on caregivers or legal representatives. These systems should be equipped to document and respect end-of-life decisions, such as preferred place of death or limitations on care, in ways that are easy to use and culturally appropriate. They should also allow the recognition of legally designated representatives, such as people holding power of attorney, to ensure that decisions are respected when individuals are no longer able to express their wishes.

Balancing access and privacy is important, particularly when third parties, such as family members or caregivers, are involved in care coordination. Health information systems must include mechanisms to define and enforce access permissions, safeguarding sensitive data while enabling the people with appropriate authority to support care and decision-making. This process is particularly relevant in the context of cognitive ability, and legal provisions for informed consent should be anticipated when individuals have full mental capacity. To support these complex decisions, ethical governance frameworks should underpin the design, implementation and use of health information systems to ensure that they always uphold the rights and dignity of older adults.

The integration of AI into health information systems introduces new ethical challenges related to healthy ageing, as these technologies may perpetuate existing ageism in society. AI may replicate the explicit and implicit biases in society based on the data input to train the models, including when older adults are underrepresented in the data used to train these systems.^37^ Such algorithmic biases can disadvantage older populations, reinforcing stereotypes and prolonging inequities due to the lack of specific and relevant outcomes for older people. Additionally, the scale and complexity of AI increase the potential for data privacy breaches and misuse. These risks underscore the need for strong ethical oversight, proactive bias mitigation strategies, and the inclusion of older adults in the design and evaluation of AI used in health information systems. As well as these safeguards, governance frameworks and regulations are needed that empower and work with older people.^37^

## Implementation challenges

Transforming health information systems for healthy ageing means navigating a range of practical, financial and ethical challenges that vary widely across contexts. Fragmentation in health, social and long-term care systems, disparities in digital literacy in older adults and providers, and uneven institutional capacity, particularly in public and private sectors, are barriers to achieving an integrated, person-centred approach. Addressing these constraints calls for realistic, phased strategies that build on existing structures and resources rather than assuming a fully digital or interoperable environment from the outset.

In resource-constrained or fragmented systems, including many low- and middle-income countries and even some high-income contexts, health and social care often have parallel data streams with limited digital infrastructure and workforce capacity. A pragmatic starting point would be to strengthen foundational elements, such as governance frameworks, clear data standards and minimum data sets, that enable links across the health, social and care areas relevant to ageing. There should be alignment with the principles of interoperability and integration discussed earlier, with an emphasis on incremental steps. For example, long-term care and social service registers could be linked using unique identifiers shared across systems to gradually improve coordination and continuity of care. Capacity-building in data management and analytics is equally important, enabling health workers and caregivers to use information effectively to improve planning and service delivery.

In many settings, older adults may still lack smartphones, broadband internet or the digital skills needed to engage with electronic systems. Health information systems should therefore include low-tech and offline options, such as short message service (SMS)-based communication, printed summaries or caregiver-mediated access, to ensure inclusion regardless of digital skills. Training programmes for older adults, caregivers and front-line workers can also help reduce barriers to participation. Equitable information systems also require visibility across all sectors of care. In many countries, a significant share of services for older adults is delivered through private or community providers, often outside national reporting systems. Integrating private sector data through governance frameworks and transparent data-sharing agreements will help ensure that information on health and social care is complete and representative of all population groups, thereby reducing bias and improving accountability of all sectors.

To implement robust privacy and data protection safeguards, countries can start by establishing incremental governance mechanisms. For example, they can first create clear data-sharing agreements and consent protocols, and progressively develop comprehensive ethical frameworks that reflect national values and legal systems. These activities should be accompanied by efforts to improve digital literacy and equitable access to enable older adults and caregivers to engage confidently and safely with digital systems.

Implementing reforms of health information systems requires sustained investment in infrastructure, digital tools and human resources. However, financing such reforms is often a challenge, including for ageing-relevant systems. For instance, funding for health information systems is frequently fragmented across disease programmes or reliant on short-term donor projects, which limits system-wide integration and long-term sustainability. Upfront costs for infrastructure, software and training can also be substantial, while recurrent expenses, such as system maintenance, data storage and workforce development, can be difficult to sustain without dedicated budget lines. To start adopting ageing-relevant health information systems, countries can undertake phased implementation. They can start with foundational, high-impact actions, for example, digitizing existing long-term care or social services registries; introducing interoperable templates that capture ageing-relevant data within current health management information systems; improving data completeness; harmonizing definitions; and piloting digital tools in priority areas such as long-term or community care. Sustaining and scaling up these efforts nationally will require additional investment and coordination. Collaborations with development partners and the private sector can help mobilize additional resources, provided that strong governance mechanisms ensure transparency, equity and public accountability.

Although the core principles presented, such as comprehensive, person-centred data, interoperability, user-centred design and ethical governance, are universally relevant, implementation of these principles must be context-specific. Countries will need to adapt these principles to their institutional capacity, digital maturity and governance structures, taking gradual, coordinated steps that reflect national realities. By doing so, countries can progressively reach health information systems that support healthy ageing, enhance equity, and ensure that no older adult is left behind.

## Conclusion

Health information systems need to be adapted to include healthy ageing. Countries should prioritize: (i) strengthening governance and interoperability across health, long-term care and social services to enable integrated care and coordinated support for older adults; (ii) adopting minimum ageing-relevant data sets, shared identifiers and analytic capabilities to ensure person-centred continuity of care; (iii) investing in workforce capacity for ageing-responsive health and social care, and enhancing digital literacy among providers and older adults; (iv) ensuring user-centred design and accessibility that reflect the needs, capacities and digital literacy of older adults, and promoting use of health information systems; (v) embedding ethical and privacy safeguards that uphold dignity and consent; and (vi) advancing stepwise, sustainably financed reforms that build on existing systems and capacity. Collectively, these actions can accelerate the transformation of health information systems into systems that deliver integrated, person-centred care that supports healthy ageing for all.
